# The Effect of Fecal Microbiota Transplantation on a Child with Tourette Syndrome

**DOI:** 10.1155/2017/6165239

**Published:** 2017-12-12

**Authors:** Huijun Zhao, Yichao Shi, Xi Luo, Lihua Peng, Yunsheng Yang, Liping Zou

**Affiliations:** ^1^Institute of Digestive Diseases, Chinese PLA General Hospital, Beijing 100853, China; ^2^Medical College of Nankai University, Tianjin 300071, China; ^3^Department of Pediatrics, Chinese PLA General Hospital, Beijing 100853, China

## Abstract

Tourette syndrome is a neuropsychiatric disorder with onset in childhood. New therapies are needed to effectively manage and treat this condition. Gut microbiota can affect central physiology and function via the microbiota-gut-brain axis. Here, we report a case in which fecal microbiota transplantation (FMT) is used to treat a child with Tourette syndrome, whose symptoms ameliorated dramatically in the following eight weeks.

## 1. Introduction

Tourette syndrome (TS) is a neurobehavioral disorder that has occurred in childhood, and it is defined by the presence of both multiple motor tics and one or more phonic tics for more than 1 year and never tic‐free for more than 3 consecutive months [1]. The main treatments for TS include behavioral treatments, administration of α2-adrenergic agonists, administration of antipsychotics, and even deep brain stimulation (DBS) [2–6]. Although current therapies may partly ameliorate tic symptoms, inadequate control of tics and adverse side effects remain challenging for the treatment of TS.

Previous studies have reported abnormalities in the cortico-basal ganglia-thalamo-cortical loops in patients with Tourette syndrome [7]. Stress, excitement, fatigue, and thermal stress can exacerbate the severity of TS, and infection with group A beta-hemolytic streptococci may induce and even aggravate tic disorders [1]. Recent research reports that gut microbiota substantially affects central physiology and function via the microbiota-gut-brain axis. Furthermore, gut microbiota plays an important role in some mental illnesses, such as depression, autism spectrum disorder, and Parkinson's disease [8]. As a result, fecal microbiota transplantation (FMT) has been considered as a potential method to rebalance gut microbiota, and its efficacy has been demonstrated in autism and epilepsy [9, 10]. Therefore, we hypothesized that gut microbiota might contribute to Tourette syndrome and assessed the effects of FMT on a child with TS. To our knowledge, this is the first report of FMT in a patient with TS.

## 2. Case Presentation

A 9-year-old boy with TS was admitted to our hospital. His symptoms predominantly consisted of involuntary eye turning, headshaking, shrugging, and voice tics, which he had experienced for 2.5 years. He was diagnosed as deficient in trace elements at a local hospital and received oral calcium tablets, with no relief of symptoms. After 6 months, the patient experienced an aggravation of the abovementioned symptoms, accompanied by trembling limbs, inattention, and memory impairments. The patient received herbal medicine (not detailed in clinical notes), but this was ineffective.

One year ago, the patient visited our hospital and was diagnosed with TS. He had a negative result for antistreptolysin O titers (ASOTs). He was prescribed tiapride 100 mg tid and probiotics, including *Bacillus subtilis*, *Clostridium butyricum*, and *Enterococcus*. In the following two months, the patient's symptoms improved. Six months ago, the tic symptoms once again increased, with the patient's total Yale Global Tic Severity Scale (YGTSS) score reaching 31, including a motor tic score of 16 and a vocal tic score of 15. The patient's parents accepted our suggestion to trial FMT to relieve the patient's tic symptoms.

A thorough assessment indicated that the patient was in a good conscious state, with normal muscle tension and physical strength. Physiological reflexes were present, and pathological reflexes were not elicited. In terms of relevant personal history, the patient had fallen downstairs and broke his head at the age of 3 years. In terms of family history, his grandfather's brother had congenital dementia. Laboratory investigations indicated no restrictions on treatment, and allergen detection did not find any inhalation allergens or food allergens. We described the treatment process of FMT and possible adverse reactions to the patient and his parents, and his father signed the consent form.

Healthy volunteers (between 10 and 40 years old) received a preliminary screening by a screening questionnaire for family history and individual disease history and then took a laboratory examination for common pathogens, including human immunodeficiency virus, hepatitis A, B, and C virus, *syphilis*, enteropathogenic *Escherichia coli* (EPEC), *Shigella*, *Salmonella*, *C. difficile* toxin, Epstein–Barr virus, cytomegalovirus, and so on, as well as fungi, ova, cysts, and parasites. Donors were not allowed to have used antibiotics, probiotics, or other agents that can influence intestinal flora before 4 weeks of donating feces. The complete screening was repeated every 3 months. The detailed screening process of donors was published in Ren et al. [11].

The fecal microbiota suspension was collected from a healthy 14-year-old male donor under rigorous screening and prepared in vitro. FMT was then administered to the patient's small intestine with 100 ml of fecal suspension via gastroscopy and to the colon with 300 ml of fecal suspension via colonoscopy under anaesthesia. There was no obvious abnormality found during endoscopy. The process of FMT treatment did not cause adverse problems, such as aspiration, vomiting, fever, abdominal pain, abdominal distension, or other adverse reactions. No abnormal clinical or biological parameters were observed before or after FMT.

Following FMT, the patient kept using tiapride 100 mg tid. His YGTSS score was the primary indicator assessed before and after FMT treatment, and we also recorded clinical evaluations and life behaviour observations of the patient (from his primary caregiver). Eight weeks after treatment, the patient's YGTSS total tic severity score decreased from 31 to 5, his motor severity score fell from 16 to 5, and his vocal severity score fell from 15 to 0, shifting from severe to mild. The boy's parents reported that the severity of their son's tic symptoms had clearly ameliorated. They reported that involuntary phonation had disappeared, and involuntary shrugging now occurred only occasionally. In addition, his attention was much more focused.

## 3. Discussion

Here, we report the case of a patient with TS, tentatively prescribed with tiapride and probiotics at first, which alleviated severity of the patient's symptoms. However, the patient experienced a relapse of symptoms, and tiapride and probiotics could not achieve remission again. Subsequently, we introduced FMT to the patient, which dramatically ameliorated his tic symptoms.

This case shows evidence for significant efficacy and safety when treating TS with FMT, suggesting that rebalancing GI microbiota might offer a promising new biological therapy for TS. However, long-term efficacy and potential adverse reactions remain unclear and warrant randomized clinical trials with large samples. The interaction between the gut and the brain remains unclear. Previous studies have indicated that the brain-gut pathway is bidirectional and that there is potentially a complex relationship between the gut microbiota and the brain [8, 12]. Further studies are urgently needed to determine whether and how gut microbiota contribute to the etiology of TS.
